# Development and Validation of a Nurse‐Specific Scale to Assess Influencing Factors in Clinical Practice Guideline Implementation: An i‐PARIHS‐Based Study

**DOI:** 10.1155/jonm/4584848

**Published:** 2026-02-01

**Authors:** Lu Liu, Namuna Dallakoti, Wei Cui, Xiangyu Li, Shu Ding, Shan Zhang

**Affiliations:** ^1^ School of Nursing, Capital Medical University, Beijing, CN 100069, China, ccmu.edu.cn; ^2^ Beijing Medical and Health Service Management and Guidance Center, Beijing Municipal Health Commission, Beijing, CN 101160, China; ^3^ Nursing Department, Beijing Anzhen Hospital, Capital Medical University, Beijing, CN 101118, China, ccmu.edu.cn; ^4^ Nursing Department, Beijing Chao Yang Hospital, Capital Medical University, Beijing, CN 100020, China, ccmu.edu.cn

**Keywords:** hospital, implementation science, nursing staff, practice guidelines, scale development, validation

## Abstract

**Background:**

Clinical practice guideline (CPG) is essential for improving the quality and consistency of care. However, gaps between guideline recommendations and nursing practice remain, contributing to adverse patient outcomes. A theory‐based tool is needed to systematically assess factors that influence nurses’ implementation of CPG.

**Objective:**

To develop and validate the *Clinical Practice Guideline Implementation Influencing Factors for Nurses* (*CPGIF-N)* scale for identifying factors affecting CPG implementation among nurses.

**Methods:**

Items were generated through a scoping review and refined via two‐round Delphi reviews. A pilot survey with 40 nurses and a cross‐sectional survey with 1005 nurses from eight tertiary hospitals were conducted. Both classical test theory (CTT) and item response theory (IRT) analyses were performed.

**Results:**

A total of 637 valid responses were analyzed. Content validity indices were high (I‐CVI: 0.93–1.00; S‐CVI/Ave: 0.99). The IRT analysis indicated that the final items demonstrated good discrimination (*a* = 1.278–4.579), ordered threshold parameters (*b*_1_ to *b*_4_), and acceptable to excellent average information (range: 0.700–2.161). Confirmatory factor analysis revealed acceptable‐fit indices (*χ* ^2^ */df = *1.956, RMSEA = 0.055, CFI = 0.935, TLI = 0.929). Average variance extracted values (0.818, 0.632, and 0.815) and composite reliability values (0.973, 0.941, and 0.982) confirmed convergent validity. The scale demonstrated excellent internal consistency (Cronbach’s *α* = 0.972; McDonald’s Omega = 0.982).

**Conclusions:**

The *CPGIF-N* scale is a reliable and valid instrument, with strong psychometric properties, offering a practical tool to evaluate factors influencing CPG implementation among nurses in China.

## 1. Background

In 2011, the Institute of Medicine (IOM) defined a clinical practice guideline (CPG) as systematically developed recommendations that evaluate evidence, weigh the benefits and risks of different interventions, and support the delivery of optimal health care [1]. The effective and standardized implementation of CPG is critical for reducing the heterogeneity and variability in clinical practice across healthcare institutions and professionals [2]. By guiding decision‐making, CPG improves patient outcomes, reduces medical errors, and decreases healthcare costs [3, 4], underscoring their broad sociological and economic significance. Within this context, growing attention has been directed toward aligning nursing practice with CPG, reflecting both the expansion of implementation science and the recognition of nursing’s central role in health service delivery [5, 6]. Nurses, as the largest group of healthcare professionals, are often the first point of contact for patients and are uniquely positioned to influence clinical outcomes [7]. Evidence supports this influence: a recent meta‐analysis [8] reported that nurse‐coordinated multidisciplinary care for heart failure reduced all‐cause mortality (relative risk: 0.80) and hospitalizations by 22% (relative risk: 0.78) [8]. Similarly, Monterosso et al. [9] found that nurse‐led interventions for cancer survivors significantly improved cognitive and social functioning while reducing fatigue. Beyond clinical benefits, nurse‐driven care models may also be cost‐effective [10]. Collectively, these findings highlight how nurses are integral to high‐quality, efficient healthcare delivery and how CPG provides an essential framework for realizing this potential.

Despite these benefits, a persistent gap remains between guideline recommendations and their implementation in clinical practice [11]. This gap can result in inefficient use of resources, variable, and, in some cases, inappropriate or harmful treatment that negatively affects their clinical outcome and prognosis [12]. Addressing the barriers and facilitators of CPG implementation is therefore vital to ensuring the adoption, sustainability, and impact of evidence‐based practice (EBP).

Yet, the dissemination of standardized nursing care derived from CPG continues to pose challenges for nurses, managers, and policy‐makers, making this a priority area of research [13, 14].

To address this, our research group conducted a scoping review [15] to summarize and synthesize findings on this topic. The review identified factors primarily related to the characteristics of CPG, healthcare professionals, hospital management, human and material resources, and policy [16, 17]. Methodologically, most existing studies rely on qualitative interviews [18] and questionnaire surveys [19]. While qualitative approaches provide valuable insights into the complexity of guideline implementation, their limited sample sizes restrict generalizability [20]. In contrast, surveys often include broader populations but rely on self‐developed instruments with limited psychometric validation, thereby reducing reliability and comparability across settings [21]. Moreover, many lack a theoretical foundation, resulting in unstructured and inconsistent identification of influencing factors [22].

One widely used instrument is the BARRIERS scale, developed by Funk et al. in 1991, which examines obstacles in translating research into practice [23]. However, its psychometric properties have been questioned. A systematic review [24] reported Cronbach’s α values for its subscales ranging from 0.47 to 0.94, with 18 of 24 studies falling below the acceptable threshold of 0.70, highlighting concerns about internal consistency and construct validity.

Given these limitations, there is a clear need for a rigorous, theoretically grounded instrument to assess the factors influencing guideline implementation in nursing practice. To address this gap, this study developed and validated the C*linical Practice Guideline Implementation Influencing Factors For Nurses* (*CPGIF-N*) scale guided by the Promoting Action on Research Implementation in Health Services Integrated Framework (i‐PARIHS). The i‐PARIHS framework was selected over the more frequently used the Consolidated Framework for Implementation Research (CFIR) because while CFIR offers a comprehensive taxonomy for identifying barriers and facilitators, it primarily serves as a diagnostic model describing what influences implementation [25]. In contrast, i‐PARIHS emphasizes how implementation occurs through facilitation and who (the facilitator) drives change by developing individual and organizational capacity [26]. This focus aligns directly with our study aim to create a tool that not only identifies influencing factors but also informs practical strategies to enhance nursing guideline implementation.

## 2. Methods

### 2.1. Conceptual Framework

**Figure 1:**
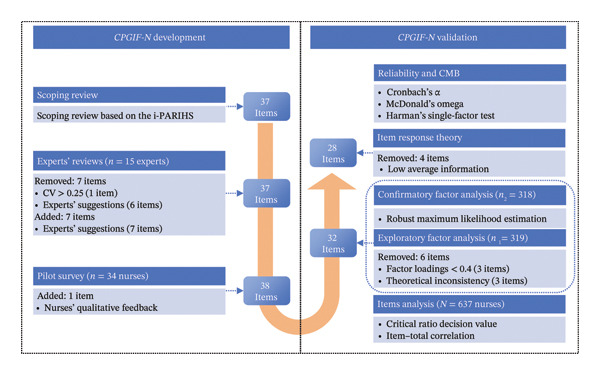
Study design process. Note: CMB = common method bias.

The i‐PARIHS framework is an evolution of the original PARIHS (Promoting Action on Research Implementation in Health Services) framework developed by the Royal College of Nursing in London [27]. It supports practitioners in evaluating the determinants of evidence uptake and facilitates successful implementation of EBP [27]. The i‐PARIHS framework is summarized in the formula **S** **I** = **Fac** ^**n**^ (**I** + **R** + **C**), where “**SI**” denotes successful implementation, “**I**” represents the innovation, “**R**” represents the recipient, “**C**” represents the context, and “Fac^n^” (facilitation) serves as the activating mechanism. Innovation refers to the content of the evidence being implemented, including its quality and applicability. Recipients are the individuals or teams influenced by and influencing the implementation process. Context captures the micro‐, meso‐, and macrolevel environments, such as organizational culture, leadership, and available resources. Facilitation is central, enabling the other constructs to interact effectively. The i‐PARIHS framework has been widely applied to guide implementation research and clarify strategies in healthcare settings [28, 29]. Accordingly, the two phases of *CPGIF-N* scale development and validation were conducted under the guidance of i‐PARIHS during 2023–2024 (Figure 1).

### 2.2. Item Generation

A top‐down strategy, informed by the i‐PARIHS framework, was adopted to ensure comprehensive coverage of relevant domains and to avoid blind spots [30]. An exploratory scoping review identified empirical studies on factors influencing healthcare professionals’ adherence to CPG, spanning guideline‐related, individual, team, institutional, and policy‐level determinants [15]. Based on this review, the research team collaboratively drafted 37 preliminary items (see Supporting file A, Table S1), mapped to the three major dimensions of i‐PARIHS.

### 2.3. Content Validity

#### 2.3.1. Expert Reviews

A two‐round Delphi study was conducted with 15 clinical nursing experts in China. The inclusion criteria were as follows: ≥ 10 years of clinical nursing experience, an intermediate or higher professional title, and at least a bachelor’s degree. Experts were selected for their pragmatic insights into guideline practice in hospital units and their prior experience with scale development. Using an online questionnaire, experts rated each item on a 4‐point Likert scale (ranging from “1 = Strongly Disagree” to “4 = Strongly Agree”) [31]. They evaluated item relevance, clarity, and dimension alignment and provided qualitative feedback. The research team revised the draft scale based on consensus and recommendations.

#### 2.3.2. Pilot Survey

A pilot survey was conducted in February and March 2024 using an anonymous e‐questionnaire to assess feasibility, clarity, and response patterns. The survey included 10 demographic questions (see Supporting file B, Table S1) and the preliminary *CPGIF-N* scale. A convenience sample of 40 nurses from two tertiary hospitals in Beijing was recruited. The inclusion criteria were as follows: (1) registered nurse, (2) ≥ 1 year of clinical experience, and (3) willingness to participate. The exclusion criteria were internships, refresher‐course trainees, or nurses on leave during data collection. Participants rated items on a 5‐point Likert scale (“1 = Totally Disagree” to “5 = Totally Agree”) and provided open‐ended feedback. Of 40 distributed surveys, 34 valid responses were analyzed, and further refinements were made to the scale.

### 2.4. Construct Validity

#### 2.4.1. Data Collection

A formal cross‐sectional survey was conducted in March–April 2024 across eight tertiary hospitals in Beijing. Questionnaires were distributed online through hospital administrators. Invalid questionnaires were excluded if (1) all items were scored as “1” (indicating nonengagement), (2) reported age was < 20 or > 55 years, or if work experience was inconsistent with age, and (3) completion time was < 3 min.

#### 2.4.2. Sample Size

A total of 1005 questionnaires were returned; 637 valid responses were retained (validity rate of 63.3%). For exploratory factor analysis (EFA), the recommended subject‐to‐variable ratio is typically 5:1 to 10:1 [32]. With 38 items in our pre‐analysis pool, the minimum sample size for EFA was 38 × 5 = 190. Accounting for an expected 15% of invalid responses according to the pilot study, the target sample size for EFA became 224. For confirmatory factor analysis (CFA), a minimum sample of *N* ≥ 200 is generally recommended [33]. Applying the same 15% allowance for invalid responses, the final target sample size for CFA was set at 236. Thus, the achieved sample was sufficient for robust factor analysis.

### 2.5. Statistical Analysis

All analysis was performed using IBM SPSS Statistics (version 25), Mplus (version 8.3), and RStudio (version 4.4.1, “mirt” package). Descriptive statistics were presented as mean ± SD or medians and interquartile ranges [*M* (*P*_25_, *P*_75_)] for continuous variables and proportions for categorical variables. Multivariate normality was tested using Mardia’s skewness and kurtosis via WebPower tools (https://webpower.psychstat.org/wiki/tools/index).

#### 2.5.1. Item Analysis

Item discrimination was examined using the critical ratio method, comparing the highest and lowest scoring 27% of participants [34]*.* Items with *t*‐values < 3.0 or *p* ≥ 0.05 were removed [35]. Item–total correlations were assessed using Pearson’s correlation coefficient (*r*); items with *r* < 0.4 were excluded [35].

#### 2.5.2. Content and Construct Validity

Expert reliability was quantified using the authority coefficient (Cr), calculated from judgment (Ca) and familiarity (Cs) indices. Consensus was assessed with Kendall’s harmony coefficient (W) and the coefficient of variation (CV). Items with CV < 0.25 were retained [36].

Content validity was determined using item‐level (I‐CVI) and scale‐level (S‐CVI/Ave) indices. Thresholds of I‐CVI ≥ 0.78 and S‐CVI/Ave ≥ 0.80 were considered acceptable [37]. For construct validity, the dataset was randomly split for EFA (*n*_1_ = 319) and CFA (*n*_2_ = 318) [38]. The Kaiser–Meyer–Olkin (KMO) measure exceeds 0.60, and Bartlett’s test of sphericity yields statistically significant results (*p* < 0.01), suggesting that a factor analysis was appropriate to perform on the data [39]. Given that the three dimensions of the i‐PARIHS framework are theoretically interrelated, parallel analysis (PA) and principal axis factoring (PAF) EFA with direct oblimin rotation were analyzed for factor retention to identify a final factor solution [40]. Factors are to be retained when the observed data eigenvalue is greater than the mean random data eigenvalue at the 95th percentile [41]. Items with factor loading values below 0.4 or cross‐loadings across multiple factors with an absolute difference in loading values of less than 0.1 were excluded [42]. CFA assessed the latent structure using model fit indices: chi‐square test/degrees of freedom ratio (*χ* ^2^ */df*) < 3.00, comparative fit index (CFI) > 0.90, Tucker‐Lewis Index (TLI) > 0.90, and root mean square error of approximation (RMSEA) ≤ 0.08 [43]. Convergent validity was confirmed if average variance extracted (AVE) > 0.5, composite reliability (CR) > 0.7, and CR > AVE [44].

Item response theory (IRT) enables researchers to evaluate the psychometric properties of measurement and optimize its potential redundant items, including the following indices: (1) an item discrimination value (α) above 0.6 is considered acceptable, indicating that the item can effectively distinguish between respondents with different levels of traits; (2) threshold parameters (*b*_1_–*b*_4_) were used to evaluate item difficulty, with the expected value range between −4 and 4; and (3) the average information, computed across the ability continuum (*θ = *−4 to 4), reaches a value of 25, which is considered superior measurement precision for the scale, while a value of 16 is acceptable [45, 46]. The graded response model (GRM), the most commonly used IRT model for polytomous items, was applied in this study [47].

#### 2.5.3. Reliability and Common Method Bias Examination

Internal consistency reliability of the overall scale and individual domains was assessed using Cronbach’s α and McDonald’s Omega (ω). Following established standards [48], values exceed 0.7 for both Cronbach’s α and ω, indicating acceptable scale reliability. Due to homogeneous item phrasing in this study, Harman’s single‐factor test was employed to assess the potential common method bias. A result exceeding the 50% threshold is considered indicative of the potential common method bias [49].

### 2.6. Ethical Approval

The study was approved by the Research Ethics Committee of Capital Medical University (Approval Number Z2023SY061). All participants were informed of the study’s purpose, procedures, and their right to withdraw at any time without penalty. Informed consent was obtained electronically prior to data collection. Anonymity and confidentiality were strictly maintained throughout the study.

## 3. Results

### 3.1. Content Validity

Fifteen nursing experts participated in the Delphi process (100% response rate). Suggestions were provided by 73% (11/15) in round one and 40% (6/15) in round two. All were female, median age 42 years (IQR: 40–51), with most having 10–20 years of hospital experience (46.7%), bachelor’s degrees (73.3%), and senior titles (73.3%) (Supporting file B, Table S2.1).

Kendall’s coefficient of concordance improved from 0.140 to 0.310 (both *p* < 0.05), reflecting increased consensus. CV values ranged 0.00–0.16; one item exceeded the 0.25 threshold (CV = 0.27) and was removed. Based on the expert input, six items were deleted and seven were added (Supporting file B, Table S2.2). I‐CVI ranged 0.93–1.00, S‐CVI/Ave reached 0.99, and expert authority indices were high (Cs = 0.89, Ca = 0.97, and Cr = 0.93). The revised scale contained 37 items.

In the pilot survey, 34 valid responses were collected from 40 nurses (validity rate: 85.0%). The feedback led to the modification of two items and addition of one new item: (1) “I think that work experience related to CPG affects adherence to guideline implementation” was revised to “I think that nursing experience has an impact on adherence to CPG implementation.” (2) “I think that the level of workload affects adherence to guideline implementation” was revised to “I think that the level of stress at work has an impact on adherence to CPG implementation.” (3) A new item was added: “I think that individual variation of patient has an impact on adherence to CPG implementation.” The formal version of the scale therefore comprised 38 items.

### 3.2. Construct Validity

A total of 637 clinical nurses completed the formal survey. The median age was 35.0 years (IQR: 30.0–41.0), and the median duration of work experience was 12.0 years (IQR: 7.0–19.5). Most were female (93.6%), held a bachelor’s degree (81.0%), and had a junior professional title (62.2%). Additionally, 44.9% had received CPG training, and 56.4% had participated in CPG‐related innovations. Demographic characteristics are presented in Table 1.

**Table 1:** Characteristics of formal survey participants (*N* = 637).

Variables	*n* (%)
Age (years)	
20–30	196 (30.8)
31–40	279 (43.8)
41–50	116 (18.2)
Over 50	46 (7.2)
Gender	
Male	41 (6.4)
Female	596 (93.6)
Educational level	
Associate degree and below	102 (16.0)
Bachelor’s degree	516 (81.0)
Master’s degree and above	19 (3.0)
Professional experience (years)	
Under 6	116 (18.2)
6–10	153 (24.0)
Over 10	368 (57.8)
Professional title	
Junior	396 (62.2)
Intermediate	222 (34.8)
Senior	19 (3.0)
Position	
Clinical research nurse	2 (0.3)
Specialist nurse	145 (22.8)
Head nurse	62 (9.7)
Other	116 (18.2)
None	312 (49.0)
Department	
Internal medicine	57 (8.9)
Surgical medicine	126 (19.8)
Emergency	14 (2.2)
Intensive care unit	223 (35.0)
Outpatient	66 (10.4)
Others	151 (23.7)
Relevant experiences	
Experience in CPG training	286 (44.9)
Experience in evidence‐based medicine/evidence‐based nursing training	186 (29.2)
Experience of participation in CPG‐based innovation in your hospital	359 (56.4)
None	252 (39.5)

Abbreviation: CPG, clinical practice guideline.

Item analysis results (Supporting file B, Table S3.1) showed significant differences between items (*p* < 0.01). Item–total correlations ranged from 0.561 to 0.826 (all *p* < 0.01), exceeding the *r* > 0.40 criterion (Supporting file B, Table S3.2); thus, no items were removed. The KMO index was 0.953, and Bartlett’s test of sphericity was significant (*χ* ^2^ *= *11284.361, *p* < 0.001), supporting factor analysis.

The PA suggested that three factors should be retained (Supporting file B, Figure 1, and Supporting file B, Table S4). Consequently, EFA was performed using a three‐factor structure. As shown in Supporting file B, Table S5, during the first round of EFA, Items 16, 17, and 19 were removed due to factor loading values < 0.4 or cross‐loading with an absolute difference of < 0.1 between the loadings. Items 18, 21, and 22 were also removed due to theoretically inconsistent loading patterns. A second‐round EFA on 32 items explained 69.0% of the total variance. Final item loadings are shown in Table 2.

**TABLE 2:** Results of exploratory factor analyses (*n*_1_ = 319).

Items	Factor loadings		
	Factor 1	Factor 2	Factor 3
Item 37	**0.869**	−0.055	−0.078
Item 38	**0.839**	−0.141	−0.073
Item 30	**0.783**	−0.173	−0.034
Item 29	**0.760**	−0.055	0.056
Item 35	**0.754**	−0.217	−0.098
Item 28	**0.753**	0.148	0.106
Item 31	**0.743**	−0.158	−0.018
Item 33	**0.706**	−0.208	−0.027
Item 24	**0.697**	−0.180	0.013
Item 32	**0.696**	−0.278	−0.021
Item 27	**0.696**	0.246	0.209
Item 36	**0.656**	−0.050	0.106
Item 26	**0.645**	0.241	0.199
Item 25	**0.639**	−0.157	0.080
Item 34	**0.613**	−0.259	0.007
Item 23	**0.490**	−0.014	0.337
Item 4	0.010	−**0.899**	0.064
Item 3	0.086	−**0.842**	0.020
Item 6	0.107	−**0.807**	0.083
Item 7	0.115	−**0.746**	0.102
Item 1	0.126	−**0.722**	0.044
Item 8	0.155	−**0.692**	0.076
Item 2	0.053	−**0.684**	0.108
Item 5	0.028	−**0.677**	0.257
Item 12	−0.014	0.013	**0.868**
Item 9	−0.147	−0.133	**0.785**
Item 11	−0.030	−0.108	**0.754**
Item 13	0.050	−0.007	**0.751**
Item 14	0.185	−0.016	**0.627**
Item 10	0.095	−0.074	**0.603**
Item 20	0.275	−0.011	**0.568**
Item 15	0.167	−0.106	**0.556**
Eigenvalues	17.295	2.847	1.940
Cumulative variance contribution rate (%)	54.047	62.943	69.007

*Note:* Factor loadings from a principal axis factoring analysis with direct oblimin rotation. Bold is the high factor load of each column, in order to determine which factor each item belongs to.

**Figure 2:**
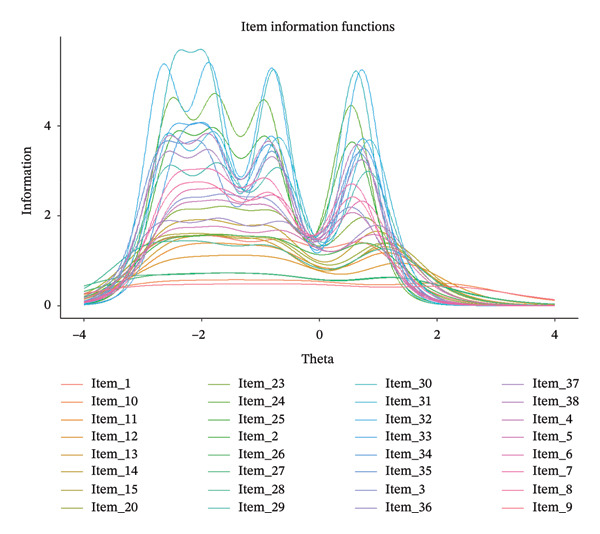
Item information functions of the 32 items.

Mardia’s test (Supporting file B, Figure 2) indicated a violation of multivariate normality (*p* < 0.001 for both skewness and kurtosis), justifying the use of robust maximum likelihood estimation (MLR) for the CFA [50]. The initial CFA model showed suboptimal fit: *χ* ^2^ */df = *3.180, RMSEA = 0.083, CFI = 0.833, and TLI = 0.821. Model modification by correlating residuals of items with large modification indices (7–8, 9–10, 26–27, 26–28, 27–28, and 37–38) improved fit to acceptable levels (*χ* ^2^ */df = *2.145, RMSEA = 0.060, CFI = 0.913, and TLI = 0.906). Items within the same group measured identical dimensions using highly similar expressions, which may introduce common method bias [51]; therefore, it was theoretically justified to allow covariance between their error terms. Three factors were identified: context (Factor 1), innovation (Factor 2), and recipient (Factor 3).

### 3.3. IRT Calibrations

IRT calibration (Supporting file B, Table S6) of the 32‐item version demonstrated good discrimination (*α* = 1.278–4.579) and ordered thresholds (*b*_1_ *<* *b*_2_ *<* *b*_3_ *<* *b*_4_). Following the established IRT practice, an average item information of 0.500 (16/32 items) was adopted as a benchmark for item retention. This threshold corresponds to the minimum average contribution per item for a total test information value of 16, which prior studies have identified as indicating acceptable measurement precision [46]. Accordingly, Items 9, 10, 26, and 27 were removed due to comparatively low information values. The item information curves (Figure 2) provide a clear visual representation of this refinement, resulting in a final 28‐item scale (see Supporting file A, Table S2).

### 3.4. Model Fit Assessment

**Figure 3:**
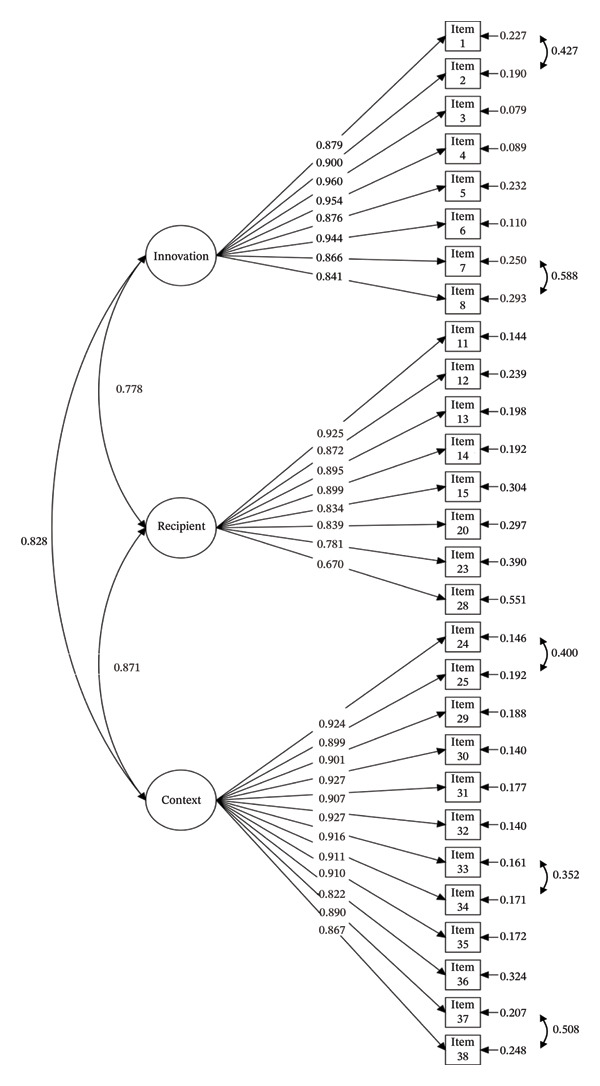
Final path diagram of standardized estimates from CFA (*n*_2_ = 318). Note. The measurement errors for Items 1–2, Items 7–8, Items 24–25, Items 33–34, and Items 37–38 were specified as a covariant relationship.

A subsequent CFA confirmed the three‐factor structure of the 28‐item scale. Initial fit indices were *χ* ^2^ */df* = 2.542, RMSEA = 0.070, CFI = 0.894, and TLI = 0.886. To improve model fit, covariances between the error terms for Items 1–2, 7–8, 24–25, 33–34, and 37–38 were allowed, based on their high modification indices and potential influence of common method bias. The model achieved good fit: *χ* ^2^ */df* = 1.956, RMSEA = 0.055, CFI = 0.935, and TLI = 0.929. The three‐factor structure (Figure 3) was consistent with the i‐PARIHS framework: innovation (Items 1–8), recipient (Items 11–15, 20, 23, and 28), and context (Items 24–25 and 29–38). AVE values (0.818, 0.632, and 0.815) and CR values (0.973, 0.941, and 0.982) confirmed convergent validity.

### 3.5. Reliability and Common Method Bias

The *CPGIF-N* achieved a strong overall Cronbach’s α of 0.972 and ω of 0.982. At the dimensional level, excellent reliability was also confirmed: innovation (Cronbach’s *α* = 0.957, *ω* = 0.973), recipient (Cronbach’s *α* = 0.930, *ω* = 0.941), and context (Cronbach’s *α* = 0.958, *ω* = 0.981). Harman’s single‐factor analysis revealed that the first factor accounted for 57.906% of the total variance, exceeding the 50% threshold.

## 4. Discussion

Guided by the i‐PARIHS framework, this study aimed to develop a theory‐driven scale to identify factors influencing nurses’ CPG implementation. The item generation process for the *CPGIF-N* scale was began with a scoping review of the available literature to identify relevant factors influencing CPG implementation among nurses. Following the scoping review, a two‐round Delphi review was conducted to achieve consensus among nursing experts on the initial *CPGIF-N* scale. This process ensured that the items were grounded in expert opinion and practical experience. The preliminary scale was then developed and tested in a pilot survey involving 34 nurses who met the predetermined criteria. Feedback from this phase led to language modifications and refinements of the scale items. Finally, a formal survey was conducted with 637 valid responses to further refine the scale items and clarify the structure of the scale.

### 4.1. Interpretation of the Results

Validity is primarily used to assess the accuracy and correctness of a scale, representing the degree to which the scale measures what it is intended to measure [36]. Regarding content validity, the I‐CVI ranged from 0.93 to 1.00, indicating good coordination and concentration among evaluators. The high S‐CVI/Ave of 0.99 suggested that the *CPGIF-N* scale had high content validity.

The PA and EFA revealed a three‐factor construct explaining 69.0% of the variance, demonstrating the scale’s sufficient capacity to identify factors influencing CPG implementation among clinical nurses [52]. CFA further validated the three‐factor structure, with acceptable model fit results, indicating that the structure of the scale generally aligned with the i‐PARIHS framework. IRT analysis provided detailed insights into item‐level performance. Specifically, all items demonstrated good discrimination (*α* = 1.278–4.579) and exhibited monotonically increasing threshold parameters (*b*_1_ to *b*_4_), confirming that each response category was meaningfully utilized across the latent trait continuum. The range of information provided by the final 28 items was 0.700–2.161, verifying the scale’s overall precision [46]. The final scale provides a superior model fit: *χ* ^2^ */df* = 1.956, RMSEA = 0.055, CFI = 0.935, and TLI = 0.929. Additionally, the AVE and CR values for all factors exceeded the recommended cutoff, thus suggesting acceptable convergent validity. The three domains identified were innovation, recipient, and context. These domains provide a comprehensive structure for understanding the multifaceted factors that influence the adoption and implementation of CPG in clinical practice.

Reliability reflects the degree of correlation and agreement between measurements [53]. In this study, the overall internal consistency was excellent (Cronbach’s *α* = 0.972), while the values for the subdimensions ranged from 0.930 to 0.958. The excellent reliability of the scale was further confirmed by a McDonald’s Omega of 0.982 for the total scale and values between 0.973 and 0.981 for the subscales.

#### 4.1.1. Domain‐1: Innovation

In the i‐PARIHS framework, innovation is defined as a change or improvement that arises from embedding new or improved knowledge, derived from research evidence, into specific practical settings [27]. This study identified three factors (Items 1–3) related to the characteristics of CPG. The quality of CPG is paramount for their successful adoption, as only high‐quality CPG can fulfill their potential value [54]. Concise and well‐structured documents facilitate adherence to CPG, while weak evidence and opaque development methods undermine guideline credibility [55, 56]. Factors related to the dissemination of innovation also influence CPG performance. A cross‐sectional study by Alkubati et al. [16] concluded that the unavailability of CPG could lead to discrepancies in nursing practice. Furthermore, guideline implementation costs (Item 5) may be context‐specific within the healthcare system of Mainland China. Under the ongoing Diagnosis‐Related Group (DRG) payment reform, hospitals face increasing fiscal constraints that influence the utilization scope and frequency of high‐cost materials and novel technologies in clinical practice [57].

#### 4.1.2. Domain‐2: Recipient

Recipients are individuals or groups who are affected by and influence the innovation, including medical and nursing staff, hospital administrators, and patients as a broad range of stakeholders. Facilitators are responsible for activating innovation implementation by assessing the characteristics of recipients. Through EFA, Items 16 (attitude toward CPG implementation), 17 (trust toward CPG), and 19 (participation in CPG training) were observed cross‐loadings. Item 16 on attitude was removed due to its conceptual overlap with self‐efficacy (Item 15). In addition, Items 18 (job satisfaction), 21 (workload), and 22 (physician attitude toward nursing guidelines) were expected to load on the recipient dimension but instead loaded on the context. Owing to sample and item similarity, the derived factor structure was potentially unstable [58]. Accordingly, despite meeting criteria for item deletion, Items 17–19 and 21–22 were retained for further validation in Supporting file B, Table S7, because of their established theoretical relevance to guideline adherence in the previous literature [59–61]. Items 9 (years of professional experience), 10 (department), 26 (attitudes of patients and their families), and 27 (behaviors of patients and their families) were observed low average information through IRT analysis. A multisite cross‐sectional study [62] found no significant association between ICU experience or ICU type and nurses’ guideline adherence. Therefore, Items 9–10 were not considered for retention. Since a study [17] by Song et al. identified passive reactions from patients or families as a barrier to nurses’ guideline adherence, Items 26 and 27 were retained in Supporting file B, Table S7. Furthermore, it is also crucial to consider how recipient behavior is shaped by the local culture. This relationship explains why multiple items belonging to recipient in this study cross‐loaded or loaded onto context factors. For instance, in China’s healthcare system, where nurses typically lack independent prescribing authority, nursing practice is heavily influenced by physicians [63]. Consequently, physicians’ attitudes toward nursing guidelines (Item 22) represent a particularly potent contextual determinant of nurses’ adherence, which may explain its empirical alignment with the context dimension rather than the theoretical recipient dimension in our analysis.

#### 4.1.3. Domain‐3: Context

The i‐PARIHS framework emphasizes the context in which innovation occurs, spanning from the micro‐ to the meso‐ and macrolevels, including local, organizational, and health system environments [27]. A culture of equal partnership, the presence of champions, and a commitment to EBP provide a favorable context for innovation by healthcare professionals [64–66]. Inadequate medical supplies are a major barrier to the implementation of CPG [59]. Not only does a material shortage hinder implementation, but a lack of human resources, leading to increased workload, also impedes CPG compliance [67]. Structural and electronic resources can enhance registered nurses’ adherence to guidelines by alleviating perceived workload [68, 69]. At the macrolevel, policy support for health services, such as integrating CPG into the health system and national budget, can significantly affect CPG delivery [70]. Notably, the predominantly public nature of China’s healthcare system means that “policy support” (Item 38) has unique contextual implications and operational mechanisms, such as fiscal backing, resource allocation, and institutional constraints. Consequently, when applying this item elsewhere, the construct of policy support must be interpreted according to local circumstances [57].

Overall, the *CPGIF-N* scale developed in this study demonstrates good reliability and validity. Compared to the widely used BARRIERS scale, the *CPGIF-N* was developed using a more rigorous methodological approach and possesses a clear measurement structure that supports a comprehensive and systematic assessment of factors influencing nurses’ guideline adherence. Furthermore, while the BARRIERS scale was developed in the 1990s, its content may no longer fully capture the realities of contemporary healthcare settings [24]. The *CPGIF-N* addressed this temporal gap by incorporating modern context, such as “digital resource support” (Item 36), which better aligns with current advancements in health information technology.

### 4.2. Limitations and Implications

This study was conducted using a well‐established theoretical framework, a sufficient sample size, and a scientific design. It demonstrated good validity and reliability, suggesting that the *CPGIF-N* scale is an effective tool for assessing factors influencing CPG implementation and bridging the gap between evidence and practice.

However, several limitations must be addressed. First, although participants were recruited from eight hospitals, our sample was limited to tertiary institutions in Beijing, which may affect the generalizability of the findings. Second, data filtering rules require further optimization for data robustness. Third, the findings may be susceptible to common method bias, potentially inflating the reliability estimates. Contributing factors include homogeneous item phrasing, respondents’ tendency to maintain consistency in their responses to questions, and a uniform data collection medium; nonetheless, the good fit of the hypothesized multifactor model (CFI = 0.935, TLI = 0.929, and RMSEA = 0.055) supports the robustness of the construct structure [71]. Finally, as factors may exhibit opposite properties in different contexts, this study did not differentiate between facilitators and barriers, a decision made to provide a concise and effective tool.

Building on these limitations, future research should (1) test the scale’s measurement invariance through formal cross‐validation studies across diverse healthcare contexts (e.g., secondary and community hospitals in different geographic regions) [72], (2) refine item design (e.g., marker variables for invalid responses screening and randomized item order) to enhance the robustness of data, (3) implement procedural and statistical controls for common method bias, such as diversifying item phrasing or introducing a theoretically unrelated marker variable to partial out its effect on structural parameters [71], and (4) examine cross‐cultural adaptation and validation to ensure generalizability across different healthcare systems.

Despite these limitations, the scale’s strong reliability and validity suggest that it may serve as a valuable tool for supporting CPG implementation in clinical settings.

## 5. Conclusions

This study successfully developed the *CPGIF-N* scale, a reliable and valid tool for identifying factors influencing CPG implementation among registered nurses in the Chinese context, thereby facilitating evidence‐based care delivery. The scale’s development process, rooted in theoretical foundations and empirical testing, ensures its applicability and effectiveness in nursing practice and research.

## Author Contributions

Lu Liu: investigation, formal analysis, data curation, conceptualization, writing–original draft, visualization, validation, and methodology.

Namuna Dallakoti: formal analysis and writing–review and editing.

Wei Cui: investigation and data curation.

Xiangyu Li: investigation and data curation.

Shu Ding: investigation and data curation.

Shan Zhang: supervision, project administration, and writing–review and editing.

## Funding

Financial support for this study was provided by the R&D Program of Beijing Municipal Education Commission (Grant No. KM202310025016).

## Disclosure

The funder played no role in the study design, data collection, data analysis, data explanation, or manuscript writing.

## Conflicts of Interest

The authors declare no conflicts of interest.

## Supporting Information

Additional supporting information can be found online in the Supporting Information section.

## Supporting information


**Supporting Information 1** Supporting file A: Full versions of the research scales.

**Supporting Information 2** Supporting file B: This file presents supporting survey findings for this study.

## Data Availability

The data that support the findings of this study are available from the corresponding author upon reasonable request.
