# Supplementary material for: Development and Validation of a Nurse‐Specific Scale to Assess Influencing Factors in Clinical Practice Guideline Implementation: An i‐PARIHS‐Based Study
Source: J Nurs Manag. 2026 Feb 1;2026:4584848. doi: 10.1155/jonm/4584848 (PMC12862181; doi:10.1155/jonm/4584848)
Supplement: Supplementary file 1 — Supporting Information 1 Supporting file A: Full versions of the research scales. [file JONM-2026-4584848-s002.docx]

**Table S1. The original 37 items.**

| **No.** | **Item Description** | **Scoring** | | | | | **Suggestions** |
| --- | --- | --- | --- | --- | --- | --- | --- |
|  |  | **1** | **2** | **3** | **4** | **5** |  |
| **Domain-1: Innovation** | | | | | | | |
| 1 | Usability of the CPG |  |  |  |  |  |  |
| 2 | Credibility of the CPG |  |  |  |  |  |  |
| 3 | Quality of the CPG |  |  |  |  |  |  |
| 4 | Clinical effect of CPG implementation |  |  |  |  |  |  |
| 5 | Cost of CPG implementation |  |  |  |  |  |  |
| 6 | Sustainability in CPG Implementation |  |  |  |  |  |  |
| 7 | Popularity of the CPG |  |  |  |  |  |  |
| 8 | Consistency between different CPGs on the same subject |  |  |  |  |  |  |
| **Domain-2: Recipient** | | | | | | | |
| 9 | Years of professional experience |  |  |  |  |  |  |
| 10 | Professional title |  |  |  |  |  |  |
| 11 | Education level |  |  |  |  |  |  |
| 12 | Level of professional knowledge |  |  |  |  |  |  |
| 13 | Level of professional skills |  |  |  |  |  |  |
| 14 | Understanding of disease |  |  |  |  |  |  |
| 15 | Self-efficacy |  |  |  |  |  |  |
| 16 | Individual competence in evidence-based practice |  |  |  |  |  |  |
| 17 | Subjective initiative |  |  |  |  |  |  |
| 18 | Attitude of CPG implementation |  |  |  |  |  |  |
| 19 | Trust of the CPG |  |  |  |  |  |  |
| 20 | Participation in CPG training |  |  |  |  |  |  |
| 21 | Work experience |  |  |  |  |  |  |
| 22 | Clinical autonomy |  |  |  |  |  |  |
| 23 | Level of workload |  |  |  |  |  |  |
| 24 | Multi-disciplinary teamwork |  |  |  |  |  |  |
| 25 | Team management |  |  |  |  |  |  |
| 26 | Department leaders’ attitude regarding CPG implementation |  |  |  |  |  |  |
| 27 | Quality of leadership within the healthcare team |  |  |  |  |  |  |
| 28 | Attitudes of patients and their families |  |  |  |  |  |  |
| **Domain-3: Context** | | | | | | | |
| 29 | Circumstance of CPG-based innovation |  |  |  |  |  |  |
| 30 | Compatibility of CPG with daily clinical routines |  |  |  |  |  |  |
| 31 | Priority of CPG implementation within a department |  |  |  |  |  |  |
| 32 | Rank of hospital |  |  |  |  |  |  |
| 33 | Effectiveness of the hospital supervision mechanisms |  |  |  |  |  |  |
| 34 | CPG training in hospitals |  |  |  |  |  |  |
| 35 | Societal allocation of healthcare resources |  |  |  |  |  |  |
| 36 | Medical insurance coverage |  |  |  |  |  |  |
| 37 | Health policy |  |  |  |  |  |  |

**Note:** CPG / CPGs= Clinical practice guideline / Clinical practice guidelines. Scoring: 1 = Totally disagree, 2 = Disagree, 3 = Not sure, 4 = Agree, 5 = Totally agree.

**Table S2. The final 28 items.**

Instructions:

We invite you to evaluate the following factors based on their influence on your adherence to clinical practice guidelines. Please mark (√) the number (1 = Totally disagree, 2 = Disagree, 3 = Not sure, 4 = Agree, 5 = Totally agree) that best matches your opinion.

| **No.** | **Item** | **Item Description** | **Scoring** | | | | |
| --- | --- | --- | --- | --- | --- | --- | --- |
|  |  |  | **1** | **2** | **3** | **4** | **5** |
| 1 | 1 | I think that **the operability of CPG** has an impact on adherence to CPG implementation. |  |  |  |  |  |
| 2 | 2 | I think that **the authority of CPG** has an impact on adherence to CPG implementation. |  |  |  |  |  |
| 3 | 3 | I think that **the quality of CPG** (e.g., clarity and evidence strength) has an impact on adherence to CPG implementation. |  |  |  |  |  |
| 4 | 4 | I think that **the clinical effect of CPG implementation** has an impact on CPG enforcement. |  |  |  |  |  |
| 5 | 5 | I think that **the cost of CPG implementation** has an impact on CPG enforcement. |  |  |  |  |  |
| 6 | 6 | I think that **the sustainability of CPG implementation** has an impact on long-term adherence. |  |  |  |  |  |
| 7 | 7 | I think that **the popularity of CPG** has an impact on adherence to CPG implementation. |  |  |  |  |  |
| 8 | 8 | I think that **consistency between different CPGs on the same subject** has an impact on adherence to CPG implementation. |  |  |  |  |  |
| 9 | 11 | I think that **the level of professional knowledge** has an impact on adherence to CPG implementation. |  |  |  |  |  |
| 10 | 12 | I think that **the level of professional skills** has an impact on adherence to CPG implementation. |  |  |  |  |  |
| 11 | 13 | I think that **the understanding of disease** has an impact on adherence to CPG implementation. |  |  |  |  |  |
| 12 | 14 | I think that **the individual competence in evidence-based practice** has an impact on adherence to CPG implementation. |  |  |  |  |  |
| 13 | 15 | I think that **self-efficacy** (one’s belief in their own ability to complete tasks) has an impact on adherence to CPG implementation. |  |  |  |  |  |
| 14 | 20 | I think that **nursing experience** has an impact on adherence to CPG implementation. |  |  |  |  |  |
| 15 | 23 | I think that **multi-disciplinary teamwork** has an impact on adherence to CPG implementation. |  |  |  |  |  |
| 16 | 24 | I think that **department leaders’ attitude regarding CPG implementation** has an impact on adherence to CPG implementation. |  |  |  |  |  |
| 17 | 25 | I think that **the quality of leadership within the healthcare team** has an impact on adherence to CPG implementation. |  |  |  |  |  |
| 18 | 28 | I think that **physician attitude towards nursing guidelines** has an impact on adherence to CPG implementation. |  |  |  |  |  |
| 19 | 29 | I think that **the circumstance of CPG-based innovation** has an impact on adherence to CPG implementation. |  |  |  |  |  |
| 20 | 30 | I think that **the compatibility of CPG with daily clinical routines** has an impact on adherence to CPG implementation. |  |  |  |  |  |
| 21 | 31 | I think that **the priority of CPG implementation within a department** has an impact on CPG enforcement. (e.g., in an emergency department, where the focus is on first aid, recommendations regarding continuous nursing may be a lower priority.) |  |  |  |  |  |
| 22 | 32 | I think that **the availability of resources for CPG implementation** have an impact on CPG enforcement. |  |  |  |  |  |
| 23 | 33 | I think that **the effectiveness of the hospital supervision mechanisms** has an impact on adherence to CPG implementation. |  |  |  |  |  |
| 24 | 34 | I think that the **CPG training in hospitals** has an impact on adherence to CPG implementation. |  |  |  |  |  |
| 25 | 35 | I think that **the awareness of hospital administrators** has an impact on adherence to CPG implementation. |  |  |  |  |  |
| 26 | 36 | I think that **the development of digital resources** (e.g., electronic health records and mobile medical equipment) has an impact on adherence to CPG implementation. |  |  |  |  |  |
| 27 | 37 | I think that **the societal allocation of healthcare resources** (e.g., distribution of supplies and staffing composition) has an impact on adherence to CPG implementation. |  |  |  |  |  |
| 28 | 38 | I think that **health policy** (e.g., health insurance policy and national health strategies) has an impact on adherence to CPG implementation. |  |  |  |  |  |

**Note:** CPG / CPGs= Clinical practice guideline / Clinical practice guidelines.
