# Supplementary material for: Development and Validation of a Nurse‐Specific Scale to Assess Influencing Factors in Clinical Practice Guideline Implementation: An i‐PARIHS‐Based Study
Source: J Nurs Manag. 2026 Feb 1;2026:4584848. doi: 10.1155/jonm/4584848 (PMC12862181; doi:10.1155/jonm/4584848)
Supplement: Supplementary file 2 — Supporting Information 2 Supporting file B: This file presents supporting survey findings for this study. [file JONM-2026-4584848-s001.docx]

| **Age (years):**______ | **Gender:** male / female | **Hospital:**_______ |
| --- | --- | --- |
| **Department:**  Internal medicine/ Surgical medicine / Emergency/ Intensive care unite/ Outpatient/ Others | | |
| **Education level:**  Associate degree and below/ Bachelor’s degree/ Master’s degree and above | | |
| **Professional title:**  Junior/ Intermediate/ Senior | | |
| **Position:**  Clinical research nurse/ Specialist nurse/ Nurse instructor/ Other; None | | |
| **Professional experience (years):**______ | | |
| **Relevant experiences:**  Experience of CPG training/ evidence-based medicine (evidence-based nursing training)/ participation in CPGs-based innovation in your hospital; None | | |
| **Work stress level:**______ (1-10 points) | | |

**Table S1.** General Information of Nurses

**Note:** CPG = Clinical practice guideline.

**Table S2.1.** Characteristics of experts panel (N=15)

| **Characteristics** | | **n (%)** |
| --- | --- | --- |
| **Age (years)** | 35-40 | 4 (26.7%) |
|  | 41-50 | 7 (46.7%) |
|  | 51-55 | 2 (13.3%) |
|  | Over 55 | 2 (13.3%) |
| **Professional experience (years)** | 10-20 | 7 (46.7%) |
|  | 21-30 | 5 (33.3%) |
|  | Over 30 | 3 (20%) |
| **Education level** | Bachelor’s degree | 11 (73.3%) |
|  | Master’s degree and above | 4 (26.7%) |
| **Professional title** | Intermediate | 4 (26.7%) |
|  | Senior | 11 (73.3%) |
| **Research interests** | Internal medicine nursing | 2 (13.3%) |
|  | Surgical nursing | 2 (13.3%) |
|  | Intensive and critical care nursing | 9 (13.3%) |
|  | Chronic diseases nursing | 1 (6.7%) |
|  | Psychiatric nursing | 1 (6.7%) |
|  | Nursing management | 5 (33.3%) |
| **Postgraduate tutor** |  | 7 (46.7%) |

**Table S2.2.** Items Alteration in Experts Reviews

| **Domain** | **Exclusion (*n*=7)** | **Domain** | **Addition (*n*=7)** |
| --- | --- | --- | --- |
| Recipient | Clinical autonomy (CV=0.27) | Recipient | Department |
| Recipient | Professional title | Recipient | Acceptance of physicians on nursing guidelines |
| Recipient | Team management | Recipient | Job satisfaction |
| Recipient | Subjective initiative | Recipient | Behaviours of patients and their families |
| Recipient | Education level | Context | Awareness of hospital administrators |
| Context | Rank of hospital | Context | Digital resources development |
| Context | Medical insurance coverage | Context | Availability of resources for CPG implementation |

**Note:** CPG = clinical practice guideline.

**Table S3.1.** Results of Critical Ratio Decision Value (N = 637)

|  | Independent Samples Test | | | | | | | | | |
| --- | --- | --- | --- | --- | --- | --- | --- | --- | --- | --- |
| Item |  | Levene's Test for Equality of Variances | | t-test for Equality of Means | | | | | | |
|  |  | F | Sig. | t | df | Sig.（2-tailed） | Mean Difference | Std. Error Difference | 95% Confidence Interval of the Difference | |
|  |  |  |  |  |  |  |  |  | Lower | Upper |
| 1 | Equal variances assumed | .033 | .856 | 18.522 | 361 | .000 | 1.472 | .079 | 1.316 | 1.629 |
|  | Equal variances not assumed |  |  | 18.401 | 339.414 | .000 | 1.472 | .080 | 1.315 | 1.630 |
| 2 | Equal variances assumed | .315 | .575 | 16.842 | 361 | .000 | 1.397 | .083 | 1.234 | 1.560 |
|  | Equal variances not assumed |  |  | 16.738 | 340.686 | .000 | 1.397 | .083 | 1.233 | 1.561 |
| 3 | Equal variances assumed | 22.410 | .000 | 17.756 | 361 | .000 | 1.471 | .083 | 1.308 | 1.634 |
|  | Equal variances not assumed |  |  | 17.512 | 299.790 | .000 | 1.471 | .084 | 1.306 | 1.637 |
| 4 | Equal variances assumed | 19.024 | .000 | 17.749 | 361 | .000 | 1.455 | .082 | 1.294 | 1.616 |
|  | Equal variances not assumed |  |  | 17.494 | 296.166 | .000 | 1.455 | .083 | 1.291 | 1.619 |
| 5 | Equal variances assumed | 2.336 | .127 | 18.086 | 361 | .000 | 1.442 | .080 | 1.285 | 1.599 |
|  | Equal variances not assumed |  |  | 17.988 | 344.174 | .000 | 1.442 | .080 | 1.285 | 1.600 |
| 6 | Equal variances assumed | 2.319 | .129 | 18.834 | 361 | .000 | 1.477 | .078 | 1.323 | 1.632 |
|  | Equal variances not assumed |  |  | 18.620 | 314.201 | .000 | 1.477 | .079 | 1.321 | 1.633 |
| 7 | Equal variances assumed | 25.796 | .000 | 19.783 | 361 | .000 | 1.445 | .073 | 1.302 | 1.589 |
|  | Equal variances not assumed |  |  | 19.475 | 288.289 | .000 | 1.445 | .074 | 1.299 | 1.591 |
| 8 | Equal variances assumed | 10.118 | .002 | 19.469 | 361 | .000 | 1.437 | .074 | 1.292 | 1.582 |
|  | Equal variances not assumed |  |  | 19.239 | 311.469 | .000 | 1.437 | .075 | 1.290 | 1.584 |
| 9 | Equal variances assumed | 3.820 | .051 | 12.205 | 361 | .000 | 1.220 | .100 | 1.023 | 1.416 |
|  | Equal variances not assumed |  |  | 12.290 | 355.678 | .000 | 1.220 | .099 | 1.024 | 1.415 |
| 10 | Equal variances assumed | 2.725 | .100 | 12.413 | 361 | .000 | 1.204 | .097 | 1.013 | 1.394 |
|  | Equal variances not assumed |  |  | 12.499 | 355.625 | .000 | 1.204 | .096 | 1.014 | 1.393 |
| 11 | Equal variances assumed | .157 | .692 | 16.242 | 361 | .000 | 1.394 | .086 | 1.225 | 1.562 |
|  | Equal variances not assumed |  |  | 16.128 | 336.866 | .000 | 1.394 | .086 | 1.224 | 1.564 |
| 12 | Equal variances assumed | .205 | .651 | 14.906 | 361 | .000 | 1.353 | .091 | 1.175 | 1.532 |
|  | Equal variances not assumed |  |  | 14.878 | 355.596 | .000 | 1.353 | .091 | 1.174 | 1.532 |
| 13 | Equal variances assumed | 1.714 | .191 | 15.696 | 361 | .000 | 1.290 | .082 | 1.128 | 1.452 |
|  | Equal variances not assumed |  |  | 15.589 | 337.939 | .000 | 1.290 | .083 | 1.127 | 1.453 |
| 14 | Equal variances assumed | .010 | .921 | 17.492 | 361 | .000 | 1.348 | .077 | 1.197 | 1.500 |
|  | Equal variances not assumed |  |  | 17.389 | 342.183 | .000 | 1.348 | .078 | 1.196 | 1.501 |
| 15 | Equal variances assumed | .812 | .368 | 16.036 | 361 | .000 | 1.241 | .077 | 1.089 | 1.393 |
|  | Equal variances not assumed |  |  | 15.992 | 353.206 | .000 | 1.241 | .078 | 1.088 | 1.394 |
| 16 | Equal variances assumed | 27.772 | .000 | 19.026 | 361 | .000 | 1.325 | .070 | 1.188 | 1.462 |
|  | Equal variances not assumed |  |  | 18.739 | 291.520 | .000 | 1.325 | .071 | 1.186 | 1.464 |
| 17 | Equal variances assumed | 18.138 | .000 | 20.257 | 361 | .000 | 1.307 | .065 | 1.180 | 1.434 |
|  | Equal variances not assumed |  |  | 19.993 | 304.280 | .000 | 1.307 | .065 | 1.178 | 1.435 |
| 18 | Equal variances assumed | .624 | .430 | 14.907 | 361 | .000 | 1.184 | .079 | 1.028 | 1.340 |
|  | Equal variances not assumed |  |  | 14.853 | 350.587 | .000 | 1.184 | .080 | 1.027 | 1.341 |
| 19 | Equal variances assumed | .028 | .867 | 17.131 | 361 | .000 | 1.403 | .082 | 1.242 | 1.564 |
|  | Equal variances not assumed |  |  | 17.027 | 341.262 | .000 | 1.403 | .082 | 1.241 | 1.565 |
| 20 | Equal variances assumed | .095 | .758 | 16.943 | 361 | .000 | 1.353 | .080 | 1.196 | 1.510 |
|  | Equal variances not assumed |  |  | 16.867 | 347.678 | .000 | 1.353 | .080 | 1.195 | 1.511 |
| 21 | Equal variances assumed | 1.331 | .249 | 15.322 | 361 | .000 | 1.341 | .088 | 1.169 | 1.513 |
|  | Equal variances not assumed |  |  | 15.224 | 340.047 | .000 | 1.341 | .088 | 1.168 | 1.514 |
| 22 | Equal variances assumed | 3.715 | .055 | 17.086 | 361 | .000 | 1.314 | .077 | 1.163 | 1.465 |
|  | Equal variances not assumed |  |  | 16.963 | 336.218 | .000 | 1.314 | .077 | 1.162 | 1.466 |
| 23 | Equal variances assumed | 1.533 | .216 | 18.724 | 361 | .000 | 1.483 | .079 | 1.327 | 1.638 |
|  | Equal variances not assumed |  |  | 18.578 | 333.414 | .000 | 1.483 | .080 | 1.326 | 1.640 |
| 24 | Equal variances assumed | 32.089 | .000 | 20.333 | 361 | .000 | 1.383 | .068 | 1.250 | 1.517 |
|  | Equal variances not assumed |  |  | 20.032 | 292.990 | .000 | 1.383 | .069 | 1.247 | 1.519 |
| 25 | Equal variances assumed | 28.615 | .000 | 19.493 | 361 | .000 | 1.379 | .071 | 1.240 | 1.518 |
|  | Equal variances not assumed |  |  | 19.198 | 291.157 | .000 | 1.379 | .072 | 1.237 | 1.520 |
| 26 | Equal variances assumed | 1.289 | .257 | 10.856 | 361 | .000 | 1.071 | .099 | .877 | 1.265 |
|  | Equal variances not assumed |  |  | 10.879 | 360.946 | .000 | 1.071 | .098 | .877 | 1.264 |
| 27 | Equal variances assumed | .540 | .463 | 11.835 | 361 | .000 | 1.101 | .093 | .918 | 1.284 |
|  | Equal variances not assumed |  |  | 11.842 | 359.930 | .000 | 1.101 | .093 | .919 | 1.284 |
| 28 | Equal variances assumed | .390 | .533 | 14.552 | 361 | .000 | 1.091 | .075 | .943 | 1.238 |
|  | Equal variances not assumed |  |  | 14.494 | 349.225 | .000 | 1.091 | .075 | .943 | 1.239 |
| 29 | Equal variances assumed | .023 | .879 | 19.947 | 361 | .000 | 1.396 | .070 | 1.258 | 1.533 |
|  | Equal variances not assumed |  |  | 19.770 | 327.863 | .000 | 1.396 | .071 | 1.257 | 1.534 |
| 30 | Equal variances assumed | 10.808 | .001 | 20.909 | 361 | .000 | 1.375 | .066 | 1.246 | 1.504 |
|  | Equal variances not assumed |  |  | 20.636 | 304.116 | .000 | 1.375 | .067 | 1.244 | 1.506 |
| 31 | Equal variances assumed | .610 | .435 | 20.130 | 361 | .000 | 1.349 | .067 | 1.217 | 1.481 |
|  | Equal variances not assumed |  |  | 19.942 | 325.539 | .000 | 1.349 | .068 | 1.216 | 1.482 |
| 32 | Equal variances assumed | 9.764 | .002 | 19.538 | 361 | .000 | 1.288 | .066 | 1.158 | 1.418 |
|  | Equal variances not assumed |  |  | 19.324 | 316.495 | .000 | 1.288 | .067 | 1.157 | 1.419 |
| 33 | Equal variances assumed | 5.253 | .022 | 18.555 | 361 | .000 | 1.283 | .069 | 1.147 | 1.419 |
|  | Equal variances not assumed |  |  | 18.393 | 328.748 | .000 | 1.283 | .070 | 1.145 | 1.420 |
| 34 | Equal variances assumed | 5.418 | .020 | 18.050 | 361 | .000 | 1.284 | .071 | 1.144 | 1.424 |
|  | Equal variances not assumed |  |  | 17.884 | 326.107 | .000 | 1.284 | .072 | 1.143 | 1.425 |
| 35 | Equal variances assumed | 12.087 | .001 | 18.944 | 361 | .000 | 1.280 | .068 | 1.147 | 1.413 |
|  | Equal variances not assumed |  |  | 18.723 | 312.136 | .000 | 1.280 | .068 | 1.146 | 1.415 |
| 36 | Equal variances assumed | 5.654 | .018 | 17.213 | 361 | .000 | 1.345 | .078 | 1.191 | 1.499 |
|  | Equal variances not assumed |  |  | 17.229 | 360.244 | .000 | 1.345 | .078 | 1.192 | 1.499 |
| 37 | Equal variances assumed | 6.942 | .009 | 19.001 | 361 | .000 | 1.315 | .069 | 1.179 | 1.451 |
|  | Equal variances not assumed |  |  | 18.811 | 321.835 | .000 | 1.315 | .070 | 1.178 | 1.453 |
| 38 | Equal variances assumed | 15.412 | .000 | 18.845 | 361 | .000 | 1.302 | .069 | 1.166 | 1.438 |
|  | Equal variances not assumed |  |  | 18.600 | 304.509 | .000 | 1.302 | .070 | 1.165 | 1.440 |

**Table S3.2.** Results of Item-Total Correlation

| **Correlation** | | | | | |
| --- | --- | --- | --- | --- | --- |
| total | Pearson Correlation | 1 |  |  |  |
|  | Sig. (2-tailed) |  |  |  |  |
|  | N | 637 |  |  |  |
| **1** | Pearson Correlation | 0.694** | **20** | Pearson Correlation | 0.728** |
|  | Sig. (2-tailed) | 0.000 |  | Sig. (2-tailed) | 0.000 |
|  | N | 637 |  | N | 637 |
| **2** | Pearson Correlation | 0.692** | **21** | Pearson Correlation | 0.665** |
|  | Sig. (2-tailed) | 0.000 |  | Sig. (2-tailed) | 0.000 |
|  | N | 637 |  | N | 637 |
| **3** | Pearson Correlation | 0.740** | **22** | Pearson Correlation | 0.755** |
|  | Sig. (2-tailed) | 0.000 |  | Sig. (2-tailed) | 0.000 |
|  | N | 637 |  | N | 637 |
| **4** | Pearson Correlation | 0.737** | **23** | Pearson Correlation | 0.762** |
|  | Sig. (2-tailed) | 0.000 |  | Sig. (2-tailed) | 0.000 |
|  | N | 637 |  | N | 637 |
| **5** | Pearson Correlation | 0.734** | **24** | Pearson Correlation | 0.817** |
|  | Sig. (2-tailed) | .000 |  | Sig. (2-tailed) | 0.000 |
|  | N | 637 |  | N | 637 |
| **6** | Pearson Correlation | 0.761** | **25** | Pearson Correlation | 0.798** |
|  | Sig. (2-tailed) | 0.000 |  | Sig. (2-tailed) | 0.000 |
|  | N | 637 |  | N | 637 |
| **7** | Pearson Correlation | 0.771** | **26** | Pearson Correlation | 0.568** |
|  | Sig. (2-tailed) | 0.000 |  | Sig. (2-tailed) | 0.000 |
|  | N | 637 |  | N | 637 |
| **8** | Pearson Correlation | 0.745** | **27** | Pearson Correlation | 0.561** |
|  | Sig. (2-tailed) | 0.000 |  | Sig. (2-tailed) | 0.000 |
|  | N | 637 |  | N | 637 |
| **9** | Pearson Correlation | 0.573** | **28** | Pearson Correlation | 0.676** |
|  | Sig. (2-tailed) | 0.000 |  | Sig. (2-tailed) | 0.000 |
|  | N | 637 |  | N | 637 |
| **10** | Pearson Correlation | 0.586** | **29** | Pearson Correlation | 0.788** |
|  | Sig. (2-tailed) | 0.000 |  | Sig. (2-tailed) | 0.000 |
|  | N | 637 |  | N | 637 |
| **11** | Pearson Correlation | 0.713** | **30** | Pearson Correlation | 0.826** |
|  | Sig. (2-tailed) | 0.000 |  | Sig. (2-tailed) | 0.000 |
|  | N | 637 |  | N | 637 |
| **12** | Pearson Correlation | 0.688** | **31** | Pearson Correlation | 0.802** |
|  | Sig. (2-tailed) | 0.000 |  | Sig. (2-tailed) | 0.000 |
|  | N | 637 |  | N | 637 |
| **13** | Pearson Correlation | 0.704** | **32** | Pearson Correlation | 0.822** |
|  | Sig. (2-tailed) | 0.000 |  | Sig. (2-tailed) | 0.000 |
|  | N | 637 |  | N | 637 |
| **14** | Pearson Correlation | 0.733** | **33** | Pearson Correlation | 0.789** |
|  | Sig. (2-tailed) | 0.000 |  | Sig. (2-tailed) | 0.000 |
|  | N | 637 |  | N | 637 |
| **15** | Pearson Correlation | 0.707** | **34** | Pearson Correlation | 0.770** |
|  | Sig. (2-tailed) | 0.000 |  | Sig. (2-tailed) | 0.000 |
|  | N | 637 |  | N | 637 |
| **16** | Pearson Correlation | 0.753** | **35** | Pearson Correlation | 0.771** |
|  | Sig. (2-tailed) | 0.000 |  | Sig. (2-tailed) | 0.000 |
|  | N | 637 |  | N | 637 |
| **17** | Pearson Correlation | 0.787** | **36** | Pearson Correlation | 0.714** |
|  | Sig. (2-tailed) | 0.000 |  | Sig. (2-tailed) | 0.000 |
|  | N | 637 |  | N | 637 |
| **18** | Pearson Correlation | 0.718** | **37** | Pearson Correlation | 0.773** |
|  | Sig. (2-tailed) | 0.000 |  | Sig. (2-tailed) | 0.000 |
|  | N | 637 |  | N | 637 |
| **19** | Pearson Correlation | 0.749** | **38** | Pearson Correlation | 0.793** |
|  | Sig. (2-tailed) | 0.000 |  | Sig. (2-tailed) | 0.000 |
|  | N | 637 |  | N | 637 |

**Note:** ** = At the 0.01 level (two-tailed), the correlation is significant

**Table S4.** The parallel analysis result

| **Factors** | **Eigenvalues for Samples** | **Random eigenvalues** | **95% Random Eigenvalues** |
| --- | --- | --- | --- |
| **1** | 20.359 | 1.724 | 1.812 |
| **2** | 2.994 | 1.636 | 1.693 |
| **3** | 1.984 | 1.573 | 1.628 |
| **4** | 1.436 | 1.514 | 1.553 |

**Table S5.** Factor Loadings of Items Exclusion in EFA

| **Items** | **Factor 1** | **Factor 2** | **Factor 3** |
| --- | --- | --- | --- |
| 22. I think that **physician attitude towards nursing guidelines** has an impact on adherence to CPG implementation. | **.721** | **-** | **-** |
| 18. I think that **job satisfaction** has an impact on adherence to CPG implementation. | **.495** | **-** | **-** |
| 21. I think that **the level of stress at work** has an impact on adherence to CPG implementation. | **.452** | **-** | **-** |
| 17. I think that **trust towards CPG** has an impact on adherence to CPG implementation. | **.354** | **-** | **-** |
| 1. I think that **attitude towards CPG implementation** has an impact on adherence to CPGs implementation. | **.350** | **-** | **-** |
| 19. I think that **participation in CPG training** has an impact on adherence to CPG implementation. | **-** | **-** | **.313** |

**Note:** “-” < 0.400; CPG = Clinical practice guideline.

**Table S6.** Item parameters: discrimination, difficulty parameters (*b*), and average information

| **Item** | **Discrimination** | **Threshold** | | | | **Average information** |
| --- | --- | --- | --- | --- | --- | --- |
|  | ***α*** | ***b_1_*** | ***b_2_*** | ***b_3_*** | ***b_4_*** |  |
| **Item 1** | 2.326 | -2.652 | -1.749 | -0.609 | 0.732 | 0.894 |
| **Item 2** | 2.329 | -2.589 | -1.666 | -0.736 | 0.813 | 0.890 |
| **Item 3** | 2.939 | -2.410 | -1.632 | -0.857 | 0.552 | 1.170 |
| **Item 4** | 2.857 | -2.431 | -1.678 | -0.842 | 0.585 | 1.132 |
| **Item 5** | 2.489 | -2.560 | -1.668 | -0.595 | 0.948 | 0.995 |
| **Item 6** | 3.033 | -2.381 | -1.653 | -0.729 | 0.733 | 1.245 |
| **Item 7** | 3.279 | -2.467 | -1.795 | -0.880 | 0.559 | 1.365 |
| **Item 8** | 3.090 | -2.480 | -1.821 | -0.828 | 0.582 | 1.267 |
| **Item 9** | 1.278 | -2.977 | -1.401 | -0.014 | 2.903 | 0.411 |
| **Item 10** | 1.388 | -2.782 | -1.449 | -0.124 | 2.084 | 0.462 |
| **Item 11** | 2.155 | -2.455 | -1.670 | -0.744 | 1.168 | 0.797 |
| **Item 12** | 1.932 | -2.528 | -1.552 | -0.668 | 1.382 | 0.700 |
| **Item 13** | 2.279 | -2.485 | -1.749 | -0.899 | 1.135 | 0.849 |
| **Item 14** | 2.561 | -2.535 | -1.776 | -0.815 | 0.996 | 1.012 |
| **Item 15** | 2.355 | -2.748 | -1.888 | -0.816 | 1.184 | 0.933 |
| **Item 20** | 2.346 | -2.653 | -1.720 | -0.758 | 1.098 | 0.921 |
| **Item 23** | 2.786 | -2.472 | -1.631 | -0.753 | 0.768 | 1.124 |
| **Item 24** | 4.216 | -2.527 | -1.765 | -0.921 | 0.538 | 1.907 |
| **Item 25** | 3.810 | -2.475 | -1.771 | -0.898 | 0.557 | 1.666 |
| **Item 26** | 1.545 | -2.922 | -1.779 | -0.740 | 1.339 | 0.512 |
| **Item 27** | 1.558 | -3.243 | -1.838 | -0.752 | 1.291 | 0.524 |
| **Item 28** | 2.236 | -2.932 | -2.010 | -0.849 | 1.118 | 0.877 |
| **Item 29** | 3.448 | -2.592 | -1.715 | -0.686 | 0.832 | 1.536 |
| **Item 30** | 4.567 | -2.460 | -1.917 | -0.777 | 0.619 | 2.065 |
| **Item 31** | 3.835 | -2.605 | -1.778 | -0.684 | 0.849 | 1.756 |
| **Item 32** | 4.579 | -2.665 | -1.867 | -0.805 | 0.718 | 2.161 |
| **Item 33** | 3.851 | -2.522 | -1.885 | -0.807 | 0.741 | 1.707 |
| **Item 34** | 3.739 | -2.342 | -1.835 | -0.836 | 0.750 | 1.586 |
| **Item 35** | 3.677 | -2.689 | -1.995 | -0.803 | 0.680 | 1.638 |
| **Item 36** | 2.656 | -2.693 | -1.690 | -0.603 | 0.997 | 1.112 |
| **Item 37** | 3.595 | -2.632 | -1.851 | -0.788 | 0.715 | 1.602 |
| **Item 38** | 3.779 | -2.613 | -1.857 | -0.848 | 0.651 | 1.691 |

**Table S7.** Items retained for future study

| **Item** | **Description** |
| --- | --- |
| **17** | I think that **trust towards CPG** has an impact on adherence to CPG implementation. |
| **18** | I think that **job satisfaction** has an impact on adherence to CPG implementation. |
| **19** | I think that **participation in CPG training** has an impact on adherence to CPG implementation. |
| **21** | I think that **the level of stress at work** has an impact on adherence to CPG implementation. |
| **22** | I think that **physician attitude** towards nursing guidelines has an impact on adherence to CPG implementation. |
| **26** | I think that **the attitudes of patients and their families** has an impact on adherence to CPG implementation. |
| **27** | I think that **the behaviors of patients and their families** has an impact on adherence to CPG implementation. |

**Note:** CPG = Clinical practice guideline.

**
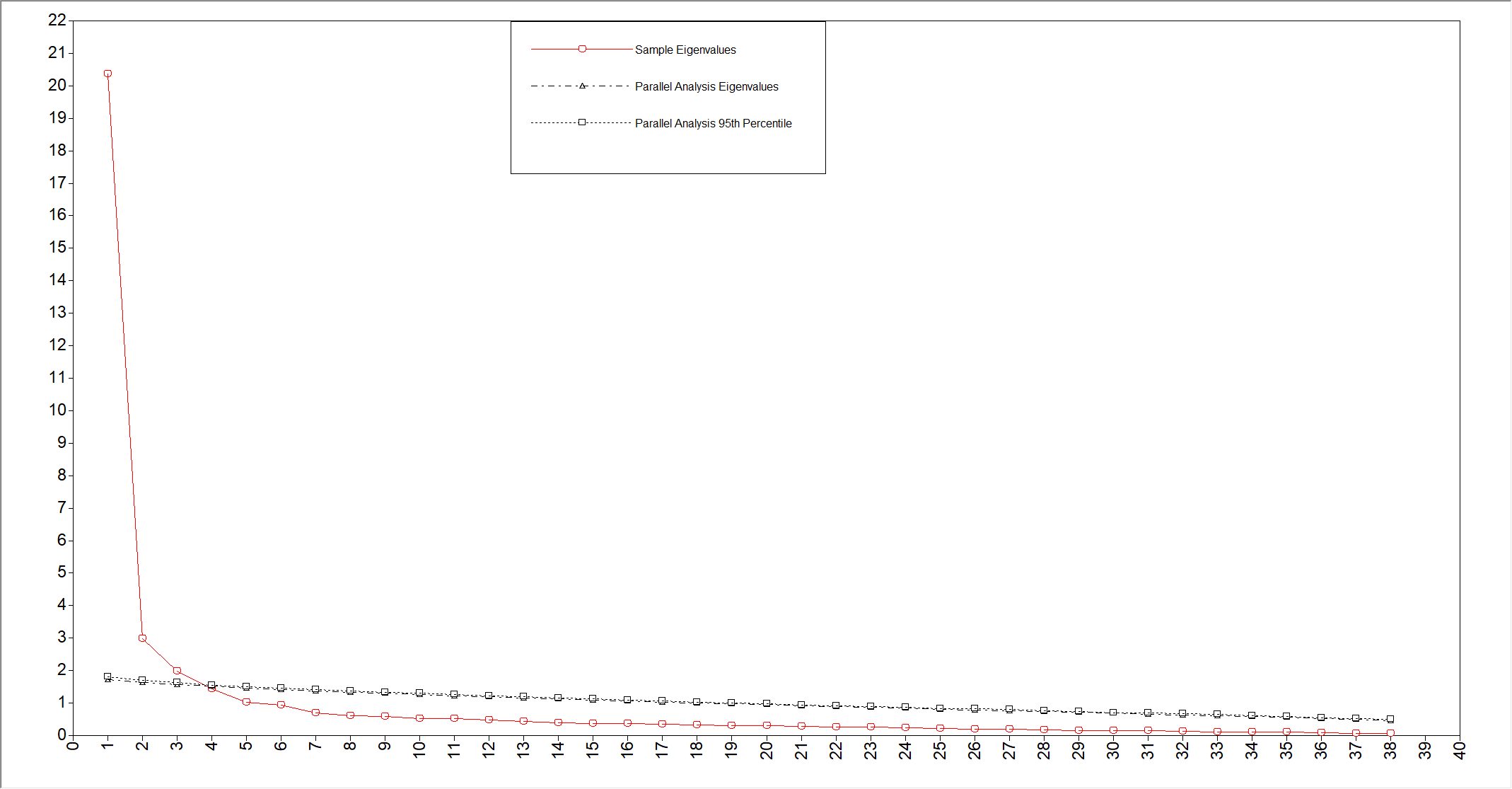
**

**Appendix Fig.1** Scree plot of PA (n_1_ = 319)


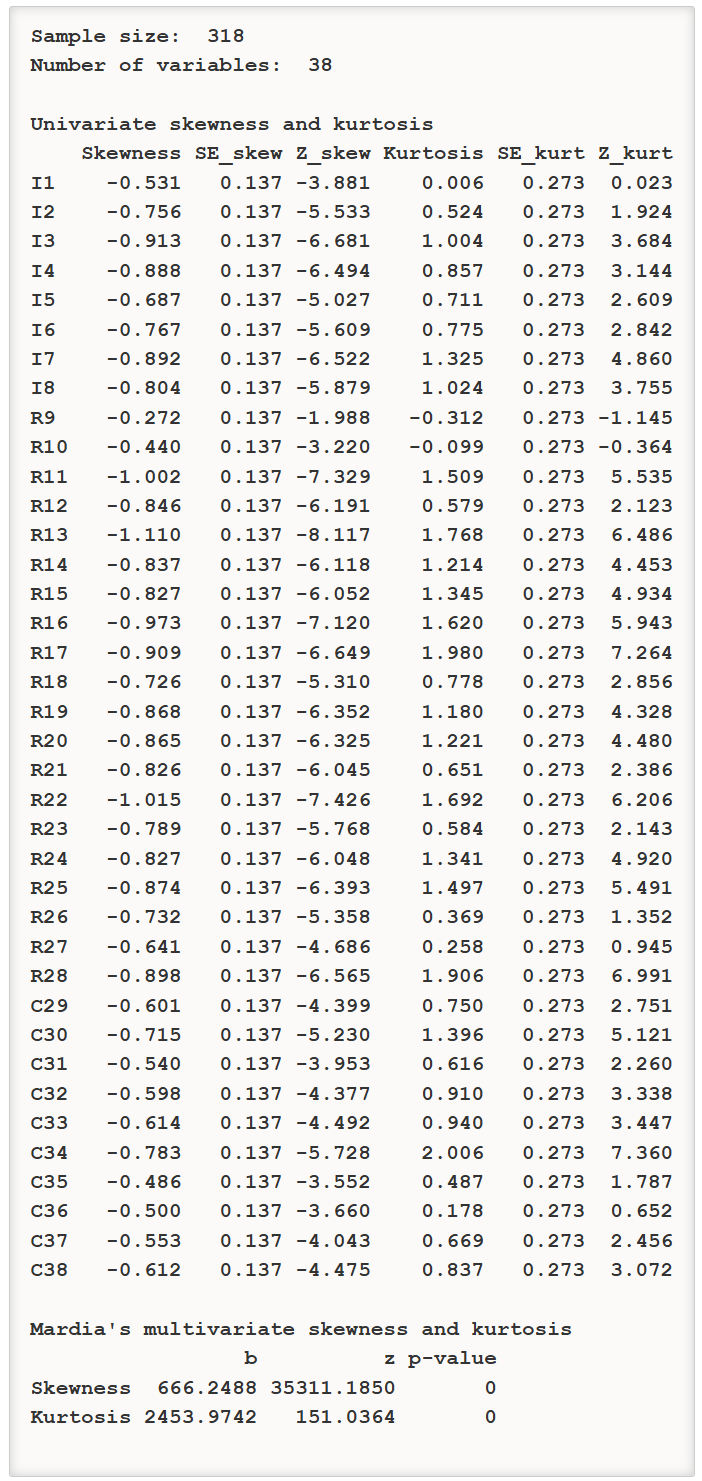


**Appendix Fig.2** The Mardia’s test results (n_2_=318)
